# Efficient Cardiovascular Parameters Estimation for Fluid-Structure Simulations Using Gappy Proper Orthogonal Decomposition

**DOI:** 10.1007/s10439-024-03568-z

**Published:** 2024-07-05

**Authors:** J. Deus, E. Martin

**Affiliations:** 1 Departamento de Ingeniería Mecánica, Máquinas y Motores Térmicos y Fluidos, Universidade de Vigo, Campus Marcosende, 36310 Vigo, Spain; 2 Instituto de Física y Ciencias Aeroespaciales (IFCAE), Universidade de Vigo, Campus de As Lagoas, 32004 Ourense, Spain

**Keywords:** Cardiovascular simulation, Windkessel model, Parameter estimation, Fluid structure interaction, Proper orthogonal decomposition

## Abstract

As full-scale detailed hemodynamic simulations of the entire vasculature are not feasible, numerical analysis should be focused on specific regions of the cardiovascular system, which requires the identification of lumped parameters to represent the patient behavior outside the simulated computational domain. We present a novel technique for estimating cardiovascular model parameters using gappy Proper Orthogonal Decomposition (g-POD). A POD basis is constructed with FSI simulations for different values of the lumped model parameters, and a linear operator is applied to retain information that can be compared to the available patient measurements. Then, the POD coefficients of the reconstructed solution are computed either by projecting patient measurements or by solving a minimization problem with constraints. The POD reconstruction is then used to estimate the model parameters. In the first test case, the parameter values of a 3-element Windkessel model are approximated using artificial patient measurements, obtaining a relative error of less than 4.2%. In the second case, 4 sets of 3-element Windkessel are approximated in a patient’s aorta geometry, resulting in an error of less than 8% for the flow and less than 5% for the pressure. The method shows accurate results even with noisy patient data. It automatically calculates the delay between measurements and simulations and has flexibility in the types of patient measurements that can handle (at specific points, spatial or time averaged). The method is easy to implement and can be used in simulations performed in general-purpose FSI software.

## Introduction

Cardiovascular diseases are the most important cause of death in the Western world [1], being this the driving reason behind the intense and active research on this topic. In recent years, interest in applying numerical simulation techniques to the study of the cardiovascular system has increasingly grown. This methodology, combined with patient-specific geometry and experimental measurements (clinical data), can assess hemodynamic behavior in a noninvasive way, aiding clinicians both in the decision-making process [2] and in personalized treatment planning [3]. It also provides enhanced capabilities in the design of medical devices [4]. In general, it can improve the understanding of the cardiovascular system function, both in health and disease conditions.

Fluid-Structure Interaction (FSI) is one of the most frequently used tools for predicting hemodynamics. It usually involves two types of analyses that are strongly coupled: (i) Computational Fluid Dynamics (CFD) to deal with the blood flow, and (ii) Solid Mechanics Finite Elements Analysis (FEA) to consider the displacements of the patient’s vessel walls surrounding the blood flow. Although significant efforts have been made in the last decade, there is no general method to solve these types of problems, and several topics are still under research from the modeling point of view, such as the constitutive laws characterizing the biological tissues and blood rheology, the boundary conditions to represent the effect of the surrounding organs, or the boundary conditions to be imposed at the inlets and outlets of the flow domain, among others [5, 6]. Regarding the latter, detailed simulation of the entire circulatory system is not computationally feasible with the current technology; therefore, detailed (full-dimensional) numerical analysis is limited to a region of particular interest, while simplified (geometrically reduced-order) models are needed to account for the effects of the omitted parts of the vascular system. These simplified models, which should provide appropriate boundary conditions upstream and downstream of the simulated region, rely on a set of free parameters that, in turn, depend on the specific physiological behavior of the patient.

In some cases, these parameters are obtained by combining literature data and manual tuning [7–9]. However, the problem is often intractable as the number of parameters increases. Furthermore, literature data values are generally averages that do not represent human variability, such as age, sex, race, and disease, to name a few examples [10–12].

Several automatic methods have been proposed to predict these parameters using different types of patient measurements. In recent years, estimations of hemodynamic parameters have largely relied on simplified models, such as zero or one dimensional (0D or 1D), which reduce the computational cost. The simplest approach to automatically tune the model parameters is to define an error functional between the simulation results and the patient measurements and use classical root-finding methods to obtain the least error parameter set. A quasi-Newton method is used in [13] to tune a total of 6 parameters of 3 different Windkessel elements using a 0D model of the abdominal aorta. Likewise, [14] employs a similar methodology to approximate 5 parameters from 4 Windkessel boundary conditions in an aortic coarctation. Due to the characteristics of the method, the Jacobian of the objective function must be calculated on each iteration, making it necessary to keep the number of objectives and model parameters small. To overcome these difficulties, in recent years, the use of data assimilation methods has gained a widespread popularity, especially the Kalman filtering family of methods. In Ref. [15], twelve Windkessel parameters were calibrated using this filtering technique in a simplified 0D model of the aorta, giving good results compared to patient measurements. These results were validated in a three-dimensional (3D) simulation with rigid walls. In Ref. [16], the Windkessel parameters were estimated in a 0D model, taking into account non-Newtonian blood flow in a carotid bifurcation using unscented Kalman filtering. Various Kalman Filter examples for the estimation of mechanical arterial properties in 1D vascular networks have been proposed, as seen in Refs. [17–19]. Kalman filtering is sequential in the sense that several parameter sets are evaluated in each time step of the numerical simulation. Unfortunately, the use of this technique is restricted to in-house software or some specific packages since most common commercial FSI simulation software cannot handle this sequential evaluation or it leads to numerical instabilities. The simplified models (0D or 1D) used in these works are accurate and fast [20]. Their main limitations are that only certain average measurements defined in certain regions of the patient’s anatomy can be used to predict the boundary condition parameters; it is not possible to introduce measurements of pressure, velocity, or any other property (e.g., wall displacement or shear stresses) at arbitrary points in the domain in the parameter estimation problem. This is particularly important because, for some situations, flow or pressure measurement cannot be obtained precisely at the zones where the simplified model is defined. In certain circumstances, it would be desirable, for example, to introduce a magnitude measured in the entire section of a vessel, such as PC-MRI measurement of velocity into the estimation process. Finally, in the cases where the parameters need to be estimated with measurements obtained in zones with complex flow patterns (near heart valves or aortic coarctation), it is not feasible to use simplified models, and employing 3D models is necessary. A few examples of parameter estimation over a complete 3D model are also available: Unscented Kalman filtering is used in Refs. [21, 22]. Windkessel parameters in an aortic dissection were predicted in Ref. [23] by utilizing an iterative optimization approach that used patient information (just the maximum and minimum pressure over the cardiac cycle) at several locations in the artery. More information about parameter estimation in blood flow modeling can be found in Ref. [24] and references therein.

This study introduces a novel parameter estimation method that exploits the properties of gappy POD to solve inverse problems. In fact, it generalizes the gappy POD procedure by leveraging the use of spatial and time averages instead of point values at some specific points in the domain. Our analysis indicates that this method exhibits comparable performance and cost to Kalman filtering, including its ability to filter noise. Also, the POD basis properties detect the time shift between pressure and flow measurement. Furthermore, the method can handle any type of patient measurements (time average, spatial average, or point measurements of volume flow, pressure, wall displacements, etc.). Additionally, the method is easy to implement and can be tuned with results simulated with commercial general-purpose software, potentially enabling its utilization in clinical practice.

First, a Reduced Order Model (ROM) is built using the g-POD methodology applied to a limited set of computed 3D-FSI solutions. These detailed numerical solutions are obtained by solving the FSI problem using different combinations of (sensible) Windkessel parameter values for the outlet boundary conditions. Then, a solution compatible with the different patient measurements at specific points/regions of the domain (e.g., time-dependent pressures and averaged flows) is reconstructed, either projecting the patient measurements over the ROM or using an optimization with constraints algorithm, where the constraints correspond to the average flow in some domain outlets. Finally, a curve fitting procedure is performed at each outlet to obtain the value of the sought parameters, and then the patient’s solution is recalculated in the entire domain and can be used as a patient prediction tool.

This method can be used with 3D simulations as well as with simplified models. In this paper, two 3D test cases (involving different 3-element Windkessel) are presented. The first one is an idealized straight artery where the parameter approximation is performed using artificial patient measurements with random noise and time delay added. The second one is an actual patient’s aorta with a mild coarctation. The problem consists in estimating a total of 12 parameters of 4 sets of 3-element Windkessel models (each of them located at an outlet of the computational domain) using clinical patient measurements.

The description of the numerical study cases used to demonstrate the potential of the tool and the methodology applied are presented in “Material and Methods” Sect. (“1·3D-FSI Numerical Model” and “2·Gappy POD Methodology” Sects., respectively), with a special mention to time shift estimation (“Estimating Measurements Time Shift” Sect.) and mean flow restrictions (“Imposed Average Flow in Multiple Outlets” Sect.). The test cases are described in “Test Cases” Sect. The results are described in “Results” Sect.: the straight artery case is presented in “Straight Artery” Sect., with the generation of the simulation database explained in “Database Generation” Sect. and the parameter estimation results in “Parameter Estimation” Sect., while the actual patient’s aorta case is presented in “Patient’s Aorta” Sect. with the description of the generation of the simulation database in “Database Generation” Sect., and the parameter estimation results in Section 3.2.2. Finally, the discussion of results and main conclusions are summarized in Section “Discussion.”

## Material and Methods

### 1·3D-FSI Numerical Model

The 3D-FSI numerical problem was solved using Ansys® Fluent 2022 R2 proprietary software for the fluid domain Ω and Ansys® Mechanical 2022 R2 for the solid domain $\Omega^{s}$. The fluid domain Ω corresponds to the internal region of the artery while the solid domain $\Omega^{s}$ denotes the volume of the aortic walls. The Navier-Stokes Eqs. (1)–(2) that model the behavior of an incompressible Newtonian fluid are solved in the fluid domain Ω:

1 $$\rho \left( {\frac{{d\vec{v}}}{dt} + \vec{v} \cdot \nabla \vec{v}} \right) = - \nabla p + \nabla \cdot \left[ {\mu \left( {\nabla \vec{v} + \left( {\nabla \vec{v}} \right)^{T} } \right)} \right] + \rho \vec{g} \quad {\text{ for }}\quad \left( {\vec{x},t} \right) \in \Omega \times \left( {0,T} \right),$$

2 $$\nabla \cdot \vec{v} = 0 \quad {\text{for}}\left( {\vec{x},t} \right) \in {\Omega } \times \left( {0,T} \right),$$

where the blood density $\rho$ is 1060 Kg/m^3^, $\vec{v}$ is the blood velocity, $p$ is the blood pressure, $\vec{g}$ is the gravity acceleration, and the blood viscosity $\mu$ is $0.004{\text{ Kg}}/\left( {\text{m s}} \right)$. $\left( {\nabla \vec{v}} \right)^{T}$ represents the transpose of the velocity gradient tensor $\nabla \vec{v}$. The Finite Volume Method (FVM) is used to solve the integral form of the differential Eqs. (1)–(2), up to the total simulated flow time $T$. For the structural analysis, Finite Element Method (FEM) is employed to solve the linear elastic shell wall Eq. (3) for the arterial domain ${\Omega }^{{\text{s}}}$:

3 $$\rho^{s} \frac{{d^{2} \vec{u}}}{{dt^{2} }} = \nabla \cdot \sigma^{s} + \vec{f}^{{\text{s}}} \quad {\text{for}}\quad \left( {\vec{x}^{s} ,t} \right) \in {\Omega }^{{\text{s}}} \times \left( {0,T} \right).$$

In Eq. (3), $\rho^{s}$ is the artery density, $\vec{u}$ is the position vector of each element of the artery, $\vec{f}^{s}$ is the external force acting on the artery wall, and $\sigma^{s}$ is the wall stress tensor, which has the form:

4 $$\sigma^{s} = C^{s} : \frac{1}{2}\left( {\nabla \vec{u} + (\nabla \vec{u}} \right)^{T} ),$$

where $C^{s}$ is a fourth-order tensor of material constants.

At the interface ${\Gamma }$ between the solid and the fluid domains, ${\Gamma } = {\Omega } \cap {\Omega }^{{\text{s}}} ,$ two conditions are satisfied: the kinematic condition:

5 $$\frac{{d\vec{u}}}{dt}\left( {x,t} \right) = \vec{v}\left( {x,t} \right) \forall \left( {x,t} \right) \in {\Gamma } \times \left( {0,{\text{T}}} \right),$$

and the force equilibrium condition:

6 $$\sigma^{s} \cdot \vec{n} = \sigma^{f} \cdot \vec{n},$$

where $\vec{n}$ is the normal vector at the boundary ${\Gamma },$ while the tensor $\sigma^{f}$ is the fluid stress tensor:

7 $$\sigma^{f} = - pI + \mu \left( {\nabla \vec{v} + \left( {\nabla \vec{v}} \right)^{T} } \right).$$

All material properties are taken from Ref. [26] except Young’s modulus, which is highly dependent on age, and a more appropriate value (equal to 0.4 MPa) was chosen from Ref. [27] assuming that the patient is a 17 years old young adult. A constant wall thickness of 1 mm is assumed.

#### Boundary and Initial Conditions

**Fig. 1:**
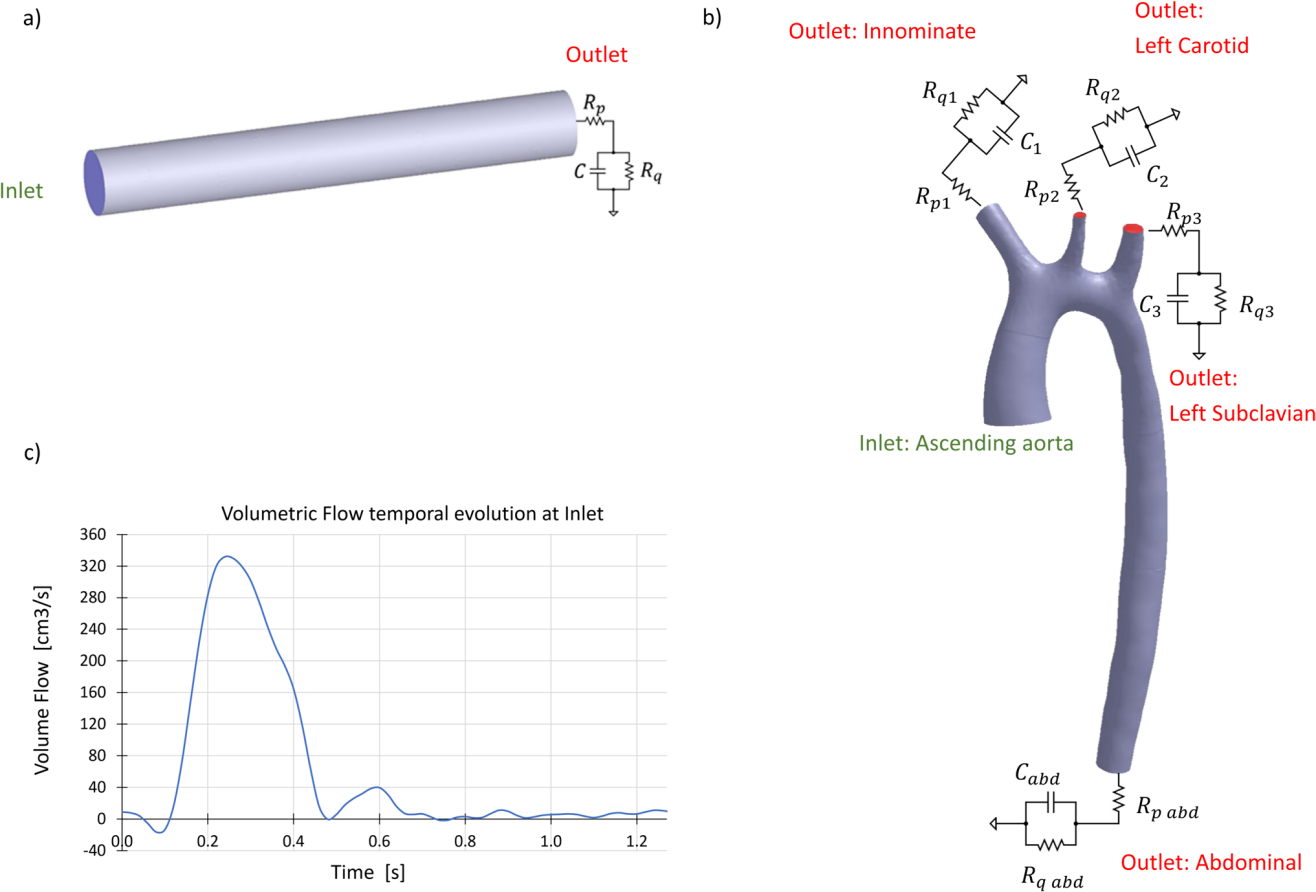
**a** Geometry and boundary conditions of the straight artery test case, **b** Geometry and boundary conditions of the patient’s aorta test case, **c** Inlet volumetric flow evolution taken from the STACOM 2013 challenge.

For the analyzed test cases (an idealized straight artery and a real patient’s aorta, described in detail in “Test Cases” Sect.), the boundary condition imposed at the inlet of the fluid domain (inlet sections shown in Fig. 1a and b) for the flow problem (1)–(2) was the temporal evolution of the patient volume flow $Q_{in} \left( t \right)$, obtained from the STACOM 2013 challenge [25] and shown in Fig. 1c.

For both test cases, the pressure outlets shown in Fig. 1a and b are modeled using 3-element Windkessel models. The Windkessel model, first proposed by Otto Frank in the early twentieth century [28], takes into account physical effects such as the resistance of the arterial tree and its compliance, which results from the fact that large arteries increase their volume when pressure is increased. For a 3-element Windkessel model, the relationship between the pressure $p$ and the volume flow $Q$ at each outlet is given by

8 $$CR_{p} \frac{\partial Q}{{\partial t}} + \left( {1 + \frac{{R_{p} }}{{R_{q} }}} \right)Q = C\frac{\partial p}{{\partial t}} + \frac{p}{{R_{q} }}.$$

Each Windkessel model depends on three parameters: the characteristic impedance $R_{p}$, which accounts for the resistance to the flow through the arteries and the phase change between pressure and flow signals, the peripheral resistance $R_{q} ,$ which accounts for the resistance of the peripheral circulation (mainly in the arterioles), and the compliance $C,$ which stands for the ratio between the volume change in the vasculature (produced by its elasticity) and the pressure change.

Equation (8) has been explicitly discretized in time using finite differences and incorporated into the software through a user-defined function. Thus, the pressure at each outlet is expressed in terms of the Windkessel parameters and the volumetric flow at the specific outlet as follows:

9 $$p^{n + 1} = p^{n} + R_{p} (Q^{n + 1} - Q^{n} ) + \frac{{{\Delta }t}}{C}\left[ {\left( {1 + \frac{{R_{p} }}{{R_{q} }}} \right)Q^{n} - \frac{{p^{n} }}{{R_{q} }}} \right].$$

Parameters $R_{p} ,R_{q} , C$ for each outlet were the subject of the identification process in both test cases.

For the solid problem defined in Eq. (3), the inlet and outlets of Fig. 1 were set as fixed, imposing a zero displacement in their boundary nodes. A combination of external pressure, elastic support, and damping were used as boundary conditions to model the mechanical properties of the tissue surrounding the artery [29]. The elastic support boundary condition is also introduced as it improves the stability of the analysis.

Finally, the initial boundary conditions for the fluid and the aortic wall domains were zero velocity and zero relative pressure, and zero surface force, respectively. To ensure that the structure and fluid start from equilibrium, the pressure of the fluid at each outlet is initialized using a time ramp during the first 0.15 s. This initial pressure is chosen to reduce the convergence time to a periodic solution.

#### Numerical Implementation

For each time step, the coupling between the two solvers (fluid flow and aortic walls) is performed iteratively: the fluid system exports the pressure at the walls, then the structural part is calculated, giving as a result the displacement at the wall, and then, this displacement is transferred back to the fluid solver, where the mesh is updated to fit the new position, and finally the fluid part is calculated. To evaluate the convergence of this coupling cycle, the root-mean square of the normalized error between two consecutive exchanged fields is calculated. The coupling cycle is performed until this metric is less than 0.001 for both exchange fields (displacements and pressures) or the maximum number of coupling iterations is reached. The temporal discretization was solved with a fixed time step of 0.02 seconds using a first-order scheme. The following discretization schemes were used: for the convective terms, a second-order upwind scheme is used for the momentum, while a second-order central difference scheme is used for the pressure. A finite element method is used to solve the structural problem considering the nonlinearity arising from large deflection effects. A sensitivity analysis to mesh and time step size was carried out to select the appropriate discretization of the problem in each test case.

### Gappy POD Methodology

**Fig.2:**
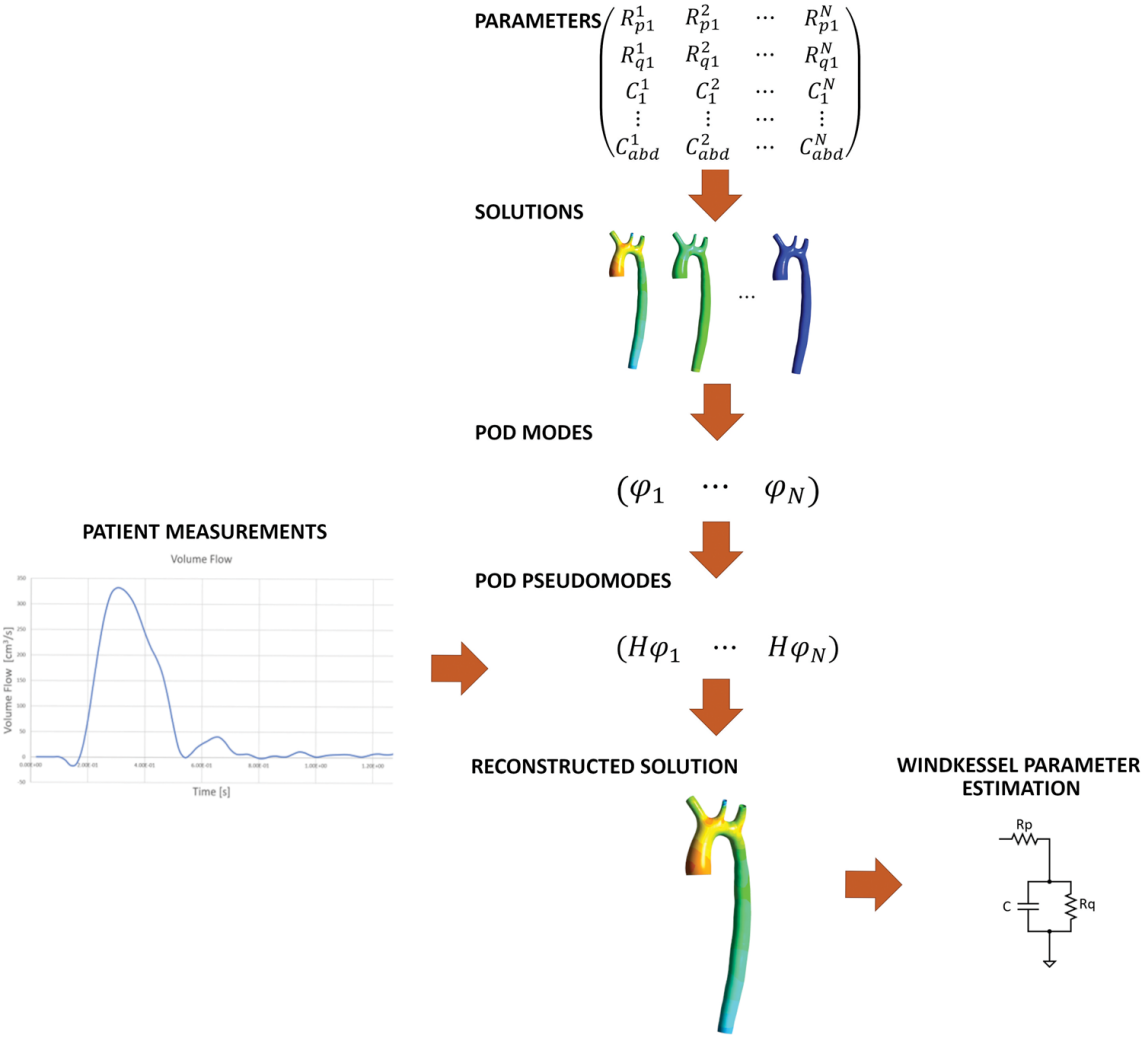
Parameter estimation with g-POD method workflow

The gappy POD technique was first introduced by Everson and Sirovich in Ref. [30] for the creation of a POD modes database with gappy (i.e., incomplete) data. It was later applied to reconstruct the aerodynamic flow around an airfoil [31]. Since then, this technique has been used to reconstruct flow fields and solve inverse problems in many contexts [32–34], including hemodynamic flows [35]. The application of the method involves two steps: the offline phase (costly in terms of computational time), and the online phase (generally fast, of less than 1 s for both studied cases). Fig. 2 shows the general parameter estimation workflow of the g-POD method.

#### Generation of the g-POD Modes

In the first phase, a set of $N$ representative snapshots ${ }\left\{ {\left. {S_{i} } \right\}_{i = 1}^{N} } \right.$ is obtained from simulations. Each snapshot $S_{i}$ is a vector (of size $M \times 1$) containing the numerical results of each computed simulation, ordered in a specific way. The complete set of snapshots, that is, the obtained numerical database $\left\{ {\left. {S_{i} } \right\}_{i = 1}^{N} } \right.$ is then stored as a matrix $S$, of size $M \times N$, which is built by stacking the $N$ snapshots consecutively in columns. Then, the POD modes $\left\{ {\left. {\varphi_{i} } \right\}_{i = 1}^{N} } \right.$ are obtained from this set of snapshots by calculating the eigenvectors (and eigenvalues) of the database correlation matrix $SS^{*}$, where * stands for the conjugate transpose operator. It can be shown that these POD modes $\varphi_{i}$ (of size $M \times 1$) are an orthonormal set of generators of the subspace spanned by the vectors of *S* and maximize the cost:

10 $$\mathop {\max }\limits_{{\varphi \in {\mathbb{R}}^{n} }} \frac{\langle{\left| {\left( {u,\varphi } \right)} \right|^{2}\rangle }}{{\left( {\varphi ,\varphi } \right)}},$$

which is equivalent to the minimization of the average projection error:

11 $$\mathop {\min }\limits_{{\varphi \in {\mathbb{R}}^{n} }} \left\langle\left\| u - \frac{{\left( {u,\varphi } \right)}}{{\left( {\varphi ,\varphi } \right)}}\varphi\right\|^{2}\right\rangle$$

where $\left\langle \cdot \right\rangle$ denotes the average operator over the entire snapshot set, $u$ is an element of S, $\left( { \cdot , \cdot } \right)$ stands for the usual dot product, $\left| \cdot \right|$ is the absolute value, and $\left\| \cdot \right\|$ stands for the usual norm. Thus, each snapshot can be represented as a linear combination of the POD modes $\varphi_{i}$ with coefficients $a_{ij} :$

12 $$S_{j} = \mathop \sum \limits_{i = 1}^{N} a_{ij} \varphi_{i}.$$

This can be thought as a data-enhanced Fourier series, so the coefficients $a_{ij}$ of the Eq. (12) can be expressed as follows:

13 $$a_{ij} = \left( {S_{j} ,\varphi_{i} } \right).$$

Because of the optimal behavior seen in Eq. (11), the snapshots can be approximated using a limited number of modes $l,$ that is, $1 \le l \le N$ that preserves the most amount of energy of the complete set of snapshots. The amount of energy retained by each mode is measured by its corresponding eigenvalue. This property allows us to build linear reduced-order models (ROMs) of the system under study, assuming that the snapshots contain enough information. An uncalculated response of the system, $\tilde{S}$, can be approximated as a linear combination of the truncated POD modes $\left\{ {\left. {\varphi_{i} } \right\}_{i = 1}^{l} } \right.$, resulting

14 $$\tilde{S} = \mathop \sum \limits_{i = 1}^{l} \alpha_{i} \varphi_{i}.$$

There are several ways to obtain the coefficients $\alpha_{i}$ of the ROM: interpolation on the snapshot’s coefficients, or a projection similar to Eq. (13), but over the available information, as in the case of g-POD.

In the common POD technique applied to this problem, the solution data for each simulated case are vectorized in $S_{i}$ stacking, for example, pressure, x-velocity, y-velocity, and z-velocity for each computational cell and time step to form the snapshot matrix. In the traditional g-POD procedure, a set of points in the computational domain is selected and the projection and identification of g-POD modes is performed only on this set. The success of the method depends on selecting the points that provide the most relevant information contained in the original snapshots.

#### Parameter Estimation Problem

Once the g-POD modes have been obtained, the next step is the estimation of the parameters. The parameter estimation problem can be seen as a stochastic dynamical system formed by a dynamical model $X_{t}$ and an observational model $Y_{t}$, where the problem is to find the appropriate set of parameters that makes the evolution of the dynamical system $X_{t}$ fit the measurements $Y_{t}$ for all times $t$, despite the errors present in both the estimation and the measurement.

In clinical cases, the available experimental measurements may be of different types. Pressure-time signals at specific locations of the patient’s aorta would fall under the traditional g-POD procedure. However, spatially integrated variables (e.g., volumetric flow vs. time at certain sections of the patient’s aorta) or spatially averaged variables (e.g., pressure–time measurements at certain sections of the patient’s aorta) are often the available clinical patient information. The method proposed in this paper generalizes the g-POD procedure not restricting the basis to the solution data available on set of points but extending its use to linear operators applied to the solution data.

Let $H$ be a linear operator that transforms from the database space to the measurement space, i.e., it transforms the stacked space and time fields into either time-dependent point measurements, flow variables surface averages, and/or volumetric flows across sections of interest^1^. In the second step of the procedure, the online phase, the measurements are first projected into the truncated POD subspace. Thus, the measurement operator *H* should be applied to the modes $\left\{ {\varphi_{i} } \right\}_{i = 1}^{l}$ to obtain a pseudo-basis of the measurement space $\left\{ {H\varphi_{i} } \right\}_{i = 1}^{l}$. Next, the coefficients $\alpha_{i}$ should be obtained. Assuming a ROM of the form of Eq. (14), we applied the $H$ operator to approximate the measurement $Y$:

15 $$Y = \mathop \sum \limits_{i = 1}^{l} \alpha_{i} H\varphi_{i}.$$

By scalar multiplication of both members of this expression with each element of the pseudo-basis, we found a system of equations of the form:

16 $$\mathop \sum \limits_{i = 1}^{l} \left( {H\varphi_{i} ,H\varphi_{j} } \right)\alpha_{i} = \left( {H\varphi_{j} ,Y} \right),$$

where $\left( { \cdot , \cdot } \right)$ stands for the usual dot product.

Once we have obtained an approximate solution using the ROM that obeys the patient measurements, the parameter estimation procedure is performed using an optimization algorithm to obtain the model parameters whose solution shows the best fit to the reconstructed solution. The problem is formulated as a nonlinear least-squares minimization, which is solved using the Trust Region Reflective algorithm, implemented in the Python library SciPy [36].

In the step of finding the pseudo-basis $\left\{ {H\left. {\varphi_{i} } \right\}_{i = 1}^{l} } \right.$ by applying the measurement operator $H$ to the POD basis $\left\{ {\left. {\varphi_{i} } \right\}_{i = 1}^{l} } \right.$, we can no longer guarantee that the set $\left\{ {H\left. {\varphi_{i} } \right\}_{i = 1}^{l} } \right.$ is orthogonal or even a basis. It will depend on the amount of information retained by the measurement operator; the problem (16) is well posed if the measurements obtained from the patient are sufficient to ensure that the reconstruction is unique. In the traditional gappy POD method, a set of points is selected, and the projection is restricted to this set. We will maintain the notation and call this restriction operator $H$. There are several techniques for selecting the minimum set that preserves the maximum amount of information. In general, the condition number of $\left( {H\varphi } \right)^{*} \left( {H\varphi } \right)$ is a measure of the retained orthonormality, being the lower the better [37]. Some sources specify a threshold of 100 as acceptable to ensure that reconstruction/inverse problems solved with g-POD have a unique solution [38]. An extension of this criterion to the measurement operators introduced earlier may be appropriate, but more research should be done to find a threshold where the measurements performed are sufficient to reconstruct the solution.

#### Estimating Measurements Time Shift

In clinical practice, the patient pressure and flow measurements are not always taken synchronously. Using the tools described above, we propose a straightforward method to predict the time shift between the two. The POD orthonormal basis is optimal in the sense that it has a maximum dot product against the entire set of snapshots, as shown in Eq. (10). If we take as a hypothesis that the input signal has most of its information in the span of the POD basis, it should have minimum approximation error when it is synchronized. Given an asynchronous measurement $Y$, we calculated the time shift $t_{S}$ using

17 $$t_{s} = {\text{arg}}\mathop {\min }\limits_{0 \le s \le P} \left(\left\| Y\left( {t + s} \right) - \mathop \sum \limits_{i = 1}^{l} \left( {Y\left( {t + s} \right),H\varphi_{i} } \right)H\varphi_{i}\right\|^{2}\right)$$

where $\left\| \cdot \right\|$ is the usual norm, and P is the cardiac cycle duration.

#### Imposed Average Flow in Multiple Outlets

In some situations, in actual medical practice, it is not possible to obtain a detailed time signal of some magnitudes but an average value. In addition, time-dependent signals may have different orders of magnitude, which can hinder the projection. To avoid these problems, we do not solve Eq. (16) to calculate the coefficients $\alpha_{i}$. Instead, we solve an optimization with restrictions problem. The cost function is the weighted error between the ROM approximations and the measurements, with the time-averaged quantities as a restriction.

The optimization problem lies in finding a set of coefficients $\alpha_{o} , \ldots ,\alpha_{l}$ that minimize the functional

18 $$\mathop \sum \limits_{j = 1}^{r} \omega_{j} \left\| \mathop \sum \limits_{i = 1}^{l} \alpha_{i} H^{j} \varphi_{i} - Y^{j}\right\|^{2}$$

where $r$ is the number of measurements, $Y^{j}$ is an experimental measurement signal, $H^{j}$ is a measure operator related to the previous measurement, and $\omega_{j}$ are weights introduced to avoid magnitude discrepancies between the different measures. This problem is restricted as follows:

19 $$\mathop \sum \limits_{i = 1}^{l} \alpha_{i} \overline{{H^{j} }} \varphi_{i} = Q^{j} 0 \le j \le K,$$

where $\overline{{H^{j} }}$ is an average measurement operator, $Q^{j}$ is an average measurement, and $j$ is the index of the measurement. We assume $0 \le j \le K$, where $K$ is the number of restrictions.

#### Test Cases

Two test cases are proposed to demonstrate the capabilities of the g-POD parameter estimation method. The first one is an idealized straight artery created with CAD software with a length of 150 mm, a diameter of 20 mm, and a wall thickness of 1 mm. The second test case is a real patient’s aorta. Both cases use the same inlet volumetric flow signal as inlet boundary condition. The real aorta geometry and patient volumetric flow measurements were obtained from the STACOM 2013 challenge [25]. The patient is a 17-year-old male with a mild thoracic aortic coarctation. Vessel geometry was obtained by magnetic resonance angiography. The geometries of both cases and the inlet flow signal^2^ are shown in Fig. 1.

The meshes for the straight artery case were formed by 1100 quad shell elements in the wall and 26480 hexahedral elements in the bulk fluid, while for the real aorta problem, 14498 quad elements, and 867240 tetrahedral elements for the solid and fluid domains, respectively, were used. The FSI simulations were run for at least 5 cardiac cycles; this ensures that a periodic solution is obtained within a relative error between equivalent points in the cardiac cycle of less than 5% for all cases. Straight artery and patient’s aorta simulations took 2 and 16 hours, respectively, on an AMD Ryzen Threadripper 2920X 3.50 GHz 12-core processor.

In both cases, the criteria used to build the numerical database were to bound a physiologically admissible region of the parameter space using a Latin hypercube strategy. In the case of the patient’s aorta, it was found that the proposed problem is not well posed because the estimation of Windkessel parameters with pressure signal and time-averaged flow can have multiple solutions. This can lead to instabilities in the optimization defined in “Imposed average flow in multiple outlets.” To avoid this problem, the simulation database is calculated close to an initial estimate of the parameter space, which is obtained by using the following steps: (a) The total resistance $R_{p} + R_{q}$ is calculated from the average pressure of the patient measurement and the average flow in each outlet. (b) For stability reasons, a value of $R_{p}$ equal to 0.05 is imposed in each Windkessel. (c) The decay factor $\gamma$ is calculated from the patient measurement by fitting the pressure decay during diastole with an exponential curve of the form $Ke^{ - \gamma t}$. Solving the Windkessel equation assuming zero flow, it can be easily seen that $\gamma = CR_{q}$. With this initial point, the snapshots were obtained following (for the parameter space) the Latin hypercube sampling strategy, multiplying or dividing each parameter by two depending on its magnitude.

## Results

### Straight Artery

#### Database Generation

**Fig. 3:**
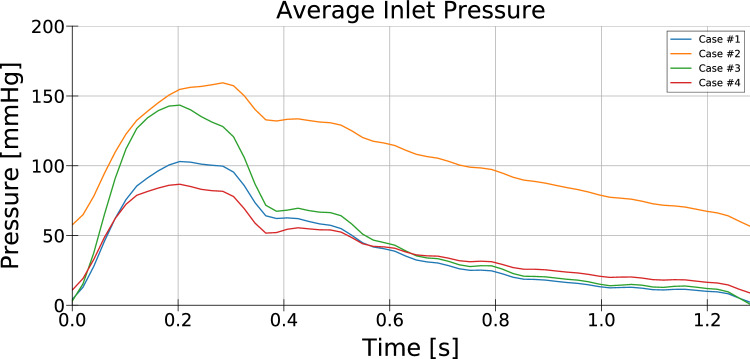
Average inlet pressure time evolution in the straight artery problem for the database cases

In the offline part of the parameter estimation procedure, a simulated database, consisting in a set of 4 cases, was computed varying the outlet Windkessel parameters $R_{p} , R_{q}$ and $C$ (see Fig. 1a). The selected simulated cases, shown in Table 1, were chosen to bound a region of the parameter space. Fig. 3 shows the obtained averaged inlet pressure for the database FSI simulated cases.

**Table 1:** Windkessel parameter values for the set of snapshots in the straight artery case

Case number	*R*_*p*_	*Rq*	*C*
#1	0.14	0.5	0.56
#2	0.14	1.5	0.56
#3	0.3	0.5	0.56
#4	0.14	0.5	1

Units for the Windkessel resistances are mm_Hg_ s/cm^3^ and cm^3^/mm_Hg_ for the compliances

**Fig. 4:**
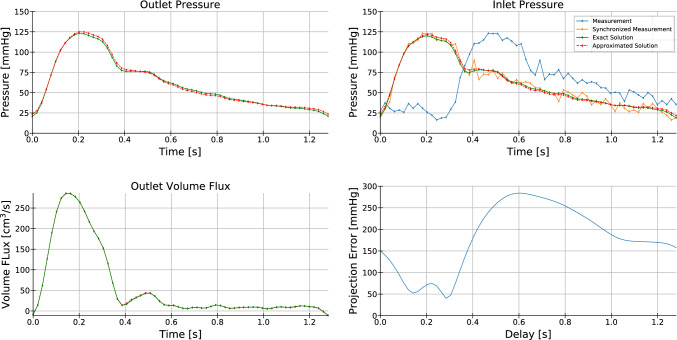
Top left: Average pressure at artery outlet for the approximated and exact solutions. Top right: Average pressure at artery inlet for the approximated and exact solutions, also the delayed and synchronized artificial measurements. Bottom left: Average volume flow at artery outlet for the approximated and exact solutions. Bottom Right: Reconstruction error against time shift

A POD basis was then constructed as described in “Material and Methods” Sect. From this basis, 3 modes were retained to build the approximate solution. The parameter estimation method was tested against a simulation with randomly selected Windkessel parameters within the region delimited by the database cases (see the selected Windkessel values of the exact numerical solution in Table 2). Patient measurements were artificially created from the exact numerical solution by adding to the inlet pressure time evolution a gaussian noise with a standard deviation of 700 mmHg and delaying the signal by 0.28 s. This artificial measurement can be seen in the top right graph of Fig. 4.

**Table 2:** Results of the parameter estimation procedure

	*R*_*p*_	*Rq*	*C*
Exact solution	0.2	0.75	0.8
Estimated parameters	0.206	0.765	0.766
Error	− 3.00%	2.11%	− 4.14%

#### Parameter Estimation

The parameter estimation was performed using the algorithm described in “Material and Methods” Sect. First, the estimation of the delay between the artificial patient measurement and the database is performed by finding the minimum of the error between the POD approximation and the patient measurement as explained in “Estimating Measurements Time Shift” Sect., this error is shown in the lower right graph of the Fig. 4, the minimum is obtained at 0.28 s, which accurately predicts the delay value. The synchronized measurement is shown in the upper right graph of Fig 4.

The solution reconstruction is now performed by calculating the pressure average of each POD mode, (i.e., applying the measurement operator $H$) to obtain the pseudo-basis where the projection is done over the synchronized measurement to obtain the reconstructed solution coefficients. In Fig. 4, a comparison between the projected and the exact solution can be seen for the outlet pressure (top left graph), for the inlet pressure (top right graph), and for the outlet volume flow (bottom left graph). Using the outlet pressure and volume flow values, a fitting algorithm is performed to obtain the parameters of the Windkessel model. The results are shown in Table 2.

### Patient’s Aorta

#### Database Generation

**Fig. 5:**
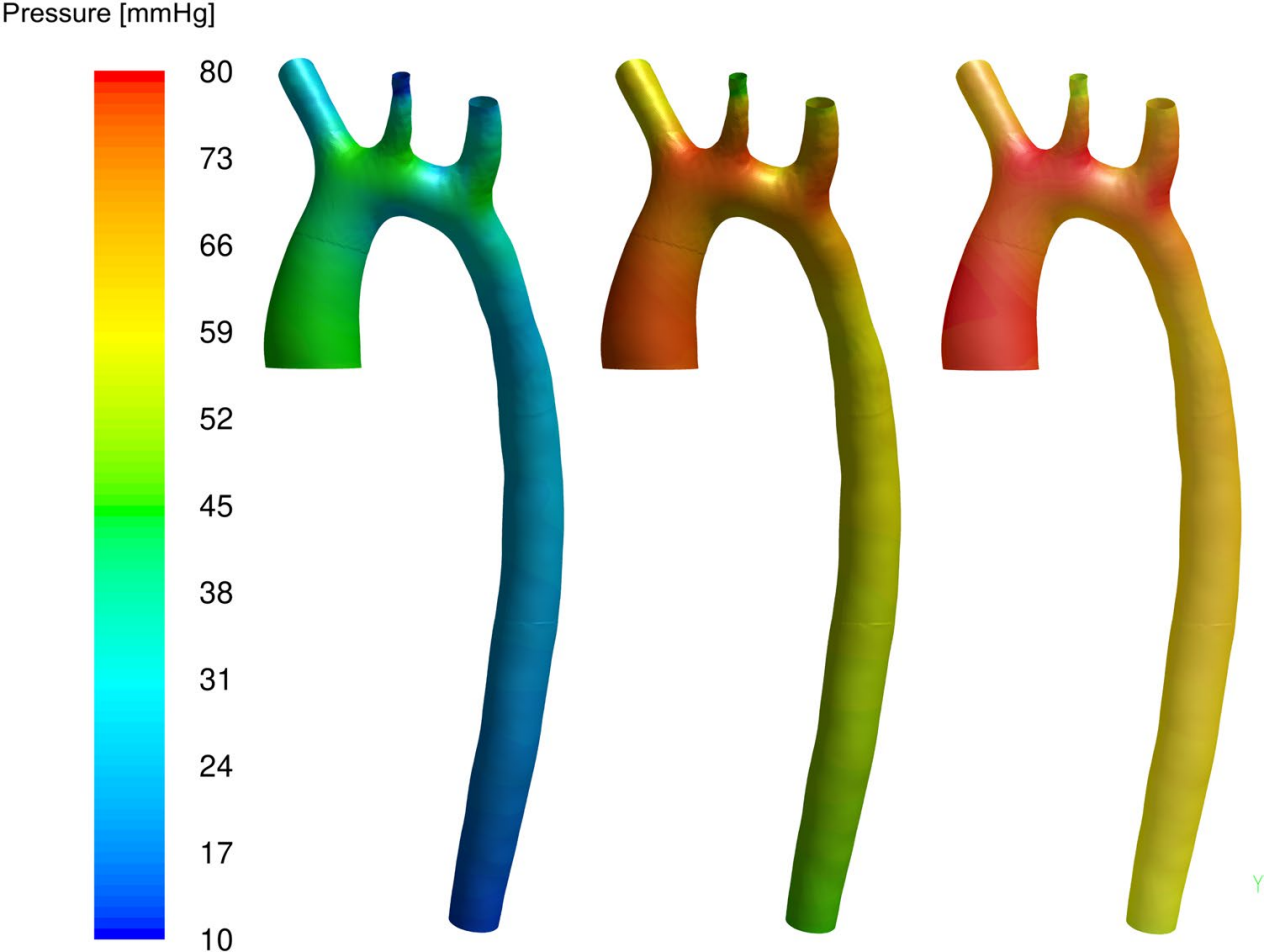
Comparison of the pressure field obtained at the aorta walls for the instants of maximum inlet pressure for cases #3, #6, and #11

For the case of the patient’s aorta, the simulation database (needed for the first offline step) was built numerically, as explained in “1·3D -FSI Numerical Model” Sect., by solving 13 different cases for the specific combinations of the 12 Windkessel parameters shown in Table 3 (see Fig. 1b for the domain location of each Windkessel parameter). This database preserves relevant information about the dependence of the flow variables (and their potential change) with the parameters of the problem. In this regard, Fig. 5 shows, for cases #3, #6, and #11, the pressure fields along the aortic walls obtained at the instants of the cardiac cycle that provided the maximum pressure at the inlet. Differences in the obtained maximum pressure are relevant, in the order of 50 %.

**Table 3:** Windkessel parameter values for the set of snapshots for patient’s aorta case

Case number	*R*_*p abd*_	*R*_*q abd*_	*C*_*abd*_	*R*_*p*1_	*R*_*q*1_	*C*_1_	*R*_*p*2_	*R*_*q*2_	*C*_2_	*R*_*p*3_	*R*_*q*3_	*C*_2_
#1	0.05	1.5	2.16	0.05	6	0.552	0.05	11.6	0.271	0.05	10	0.311
#2	0.1	1.5	2.16	0.05	6	0.552	0.05	11.6	0.271	0.05	10	0.311
#3	0.05	3	2.16	0.05	6	0.552	0.05	11.6	0.271	0.05	10	0.311
#4	0.05	1.5	4	0.05	6	0.552	0.05	11.6	0.271	0.05	10	0.311
#5	0.05	1.5	2.16	0.1	6	0.552	0.05	11.6	0.271	0.05	10	0.311
#6	0.05	1.5	2.16	0.05	3	0.552	0.05	11.6	0.271	0.05	10	0.311
#7	0.05	1.5	2.16	0.05	6	1	0.05	11.6	0.271	0.05	10	0.311
#8	0.05	1.5	2.16	0.05	6	0.552	0.1	11.6	0.271	0.05	10	0.311
#9	0.05	1.5	2.16	0.05	6	0.552	0.05	6	0.271	0.05	10	0.311
#10	0.05	1.5	2.16	0.05	6	0.552	0.05	11.6	0.5	0.05	10	0.311
#11	0.05	1.5	2.16	0.05	6	0.552	0.05	11.6	0.271	0.1	10	0.311
#12	0.05	1.5	2.16	0.05	6	0.552	0.05	11.6	0.271	0.05	5	0.311
#13	0.05	1.5	2.16	0.05	6	0.552	0.05	11.6	0.271	0.05	10	0.5

Units for the Windkessel resistances are mm_Hg_ s/cm^3^ and m^3^/mm_Hg_ for the compliances

**Fig. 6:**
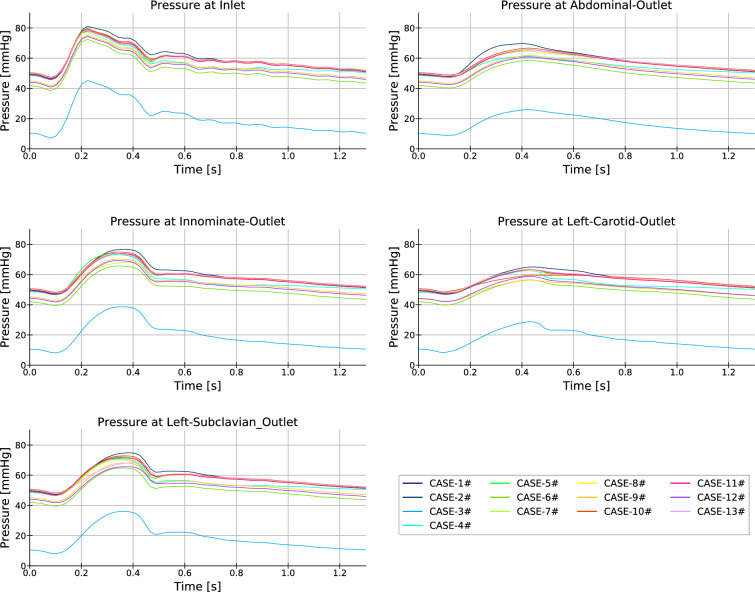
Plots of the (spatial average) pressure at the artery inlet and outlets for the different cases of the database

**Fig. 7:**
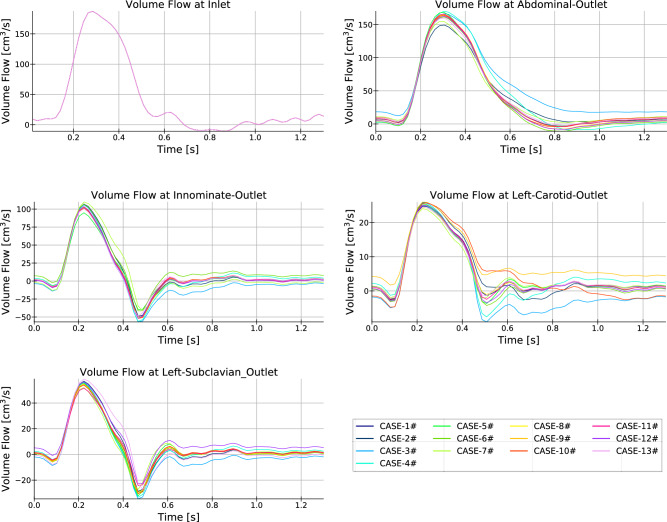
Counterpart of Fig. 6 for the volumetric flows

Fig. 6 shows the numerically obtained (spatially averaged) pressure at the different inlet and outlet sections of the computational domain for the 13 cases. Fig. 7 is the counterpart of Fig. 6 for the volumetric flows. The relative differences in these values between the different cases are relevant (the maximum variations in pressure and volumetric flows are in the range of 50% and 100%, respectively), indicating that the range of selected combinations fully covers the potential behavior of the flow.

### Parameter Estimation

**Fig. 8:**
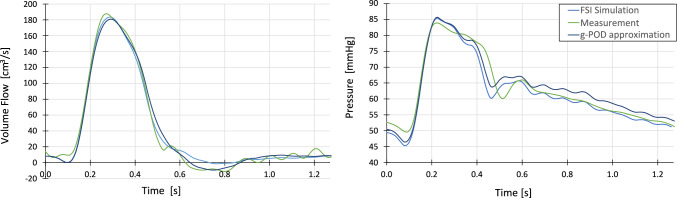
Comparison of patient measurement, g-POD approximation, and FSI simulation performed with the obtained parameters for the outlet flow and the inlet pressure

The POD basis was constructed using the numerical database previously calculated. The POD was truncated up to the first $l = 9$ modes, since 99.94% of the information was retained by them. We applied the methodology described in “Estimating Measurements Time Shift” Sect. to estimate the Windkessel parameters using the following patient measurements, obtained also from the STACOM 2013 Challenge [25]:

- Time-dependent volumetric flow measured at the abdominal outlet section (see green line in the volume flow graph of Fig. 8), obtained by phase-contrast magnetic resonance.
- Time-dependent averaged pressure at the ascending aorta inlet (green line in the pressure graph of Fig. 8), which was acquired by catheterization.
- Average flow in Innominate, Left Carotid, and Left Subclavian arteries (shown in the measurement column of Table 6).

The first step, as in the previous case, was to perform the time shift estimation, which shows a delay between patient measurements and simulations of 0.12 s for the pressure and 0.02 s for the volume flow.

In this case, the coefficients of the g-POD approximation are obtained by solving an optimization problem where the g-POD approximation error of the time-dependent volumetric flow measured at the abdominal outlet and the time-dependent averaged pressure at the ascending aortic inlet is minimized using the average flow in innominate, left carotid, and left subclavian arteries as restrictions (as seen in Imposed average flow in multiple outlets). Then, given the time-dependent average pressure and volume flow in each outlet, a curve fitting algorithm is used to obtain the parameter set. The 12 predicted Windkessel parameters that gave the best results against these measurements are shown in Table 4.

**Table 4:** Estimated Windkessel parameters

	*R*_*p*_	*R*_*q*_	*C*
Abdominal	0.0628	1.477	1.619
Innominate	0.146	6.001	0.363
Left Carotid	0.0721	11.639	0.296
Left Subclavian	0.200	10.081	0.176

Units for the Windkessel parameters are mmHg s/cm^3^ for the resistances and cm^3^/mmHg for the compliance

Fig. 8 shows the comparison between the patient measurements (time-dependent abdominal flow and time-dependent pressure at the ascending aorta inlet) and the g-POD approximation obtained from the linear combination of the selected POD modes. For comparison, a new FSI simulation (not included in the previous simulation database) was run with the estimated parameters from Table 4, and its results are also included in Fig. 8. In addition, Table 5 shows the relative L^2^ norm error between the FSI simulation and g-POD approximation, between the FSI and the patient measurements and between the g-POD reconstruction and the patient measurements. We observe that the FSI simulation accurately reproduces the g-POD approximation result.

**Table 5:** Averaged time-dependent deviations between measurement, FSI simulation, and g-POD approximation for the abdominal flow and the inlet pressure

	Abdominal volume flow (%)	Inlet pressure (%)
FSI – g-POD reconstruction	7.34	3.59
FSI – Measurement	7.99	4.70
g-POD reconstruction—Measurement	9.41	3.50

Table 6 shows the average flows obtained at the different boundaries of the problem. The optimization strategy presented in “Imposed average flow in multiple outlets” successfully imposes the outlet flow in the innominate, left carotid, and left subclavian arteries.

**Table 6:** Average flow at the different inlets and outlets and deviations from their measurement values

	Average flow [cm^3^/s]		
	Measurement	g-POD approximation	FSI Simulation
Inlet	61.83	62.01	62.54
Abdominal	40.17	40.28	40.36
Innominate	10.40	10.42	10.42
Left carotid	5.20	5.10	5.23
Left subclavian	6.07	6.21	6.15

## Discussion

A new methodology to estimate parameters (needed at the boundaries) of a FSI vascular simulation, using patient measurements in combination with gappy POD techniques, has been described and tested. Two test cases have been presented, the first consisting in the estimation of one set of parameters of a 3-element Windkessel model in an idealized aortic geometry with artificially generated patient measurements. The second one, consisting in the parameter estimation of four sets of 3-element Windkessels models of a patient’s aorta, with actual patient measurements. Both results exhibit a small deviation between the measured data and the approximated solution. For the idealized aortic case, the error in the approximated parameters is less than 4.5% for the three parameters, showing not only good agreement between the exact and reconstructed solutions (as seen in Fig. 4), but also the capabilities of POD to filter noise present in the measurements. Furthermore, the time shift estimation algorithm accurately predicts the gap between the artificial measurement and the database. In the patient’s aorta case, a relative 2-norm error of less than 8% for the flow and less than 5% for the pressure is found between the measured data and the FSI simulation run with the estimated parameters. Both the FSI simulation and the g-POD approximation show a good agreement with the measurements, although a significant delay in the dicrotic notch is observed between the FSI simulation and the patient measurement. This can be explained by differences in material properties and modeling assumptions, e.g., the stiffness of the patient’s artery and other wall boundary conditions imposed in the simulations, or problems during the measurement procedure, such as measuring pressure and volume flows at different heart rates. A peak pressure drop of 4.89 mmHg across the coarctation is found for the resting conditions. According to the literature, this value is compliant with a mild coarctation [39].

The method can be used with simplified models, 0D or 1D, as well as 3D. Although in the 3D case, the method entails the penalty of building a computationally expensive 3D-FSI simulation database, once the database for the specific patient is built, the parameters can be estimated very fast (less than 1 s) using any patient measurements (e.g., for different conditions of the patient). Once the parameters are obtained, either a direct FSI simulation can be carried out (more time consuming), or interpolation in the truncated POD basis can be done (fast approach) to obtain the patient detailed flow inside the domain (e.g., velocities, pressure, and wall stress fields). This last approach was validated in the patient’s aorta case since the error between g-POD approximation and FSI is low (7.35% for the volume flow and 3.59% for the pressure measured in relative L^2^ norm error) as seen in Fig. 8 and Table 5. Once the parameter estimation is done, more measurements of the same patient can be introduced into the process to improve the estimation result, or to avoid ill-posed problems, without recalculating the simulation database, just by redefining the measurement operator $H$. Also, additional parameter estimation can be done without recalculating the database if the geometry and the inlet volume flow remain unchanged but the other measurements had changed, for example, a change in downstream resistance or compliance.

The computational cost of the method is (N + 1) being N the number of estimated parameters, scaling linearly with N. This cost is the same as the Kalman filtering for 3D-FSI simulations reported in [21] with similar accuracy. In addition, the tests performed show that this method has some additional capabilities, such as measurement error filtering, derived from the interpretation of the POD reconstruction as a filter, and the prediction of the time shift between non-synchronous measurements.

Although the method can be used with simplified simulations, 0D or 1D, the use of fully detailed 3D numerical solutions instead of a lumped parameter model broadens the applicability of the present method as it allows to (i) obtain details of the patient fluid flow and stress behavior (not shown in the work for the sake of brevity), and (ii) work alternatively with different type of patient measurements (either spatial-averaged, time-averaged, or point values), obtained at any location inside the simulated domain, as shown in the patient’s aorta test case. Therefore, information gathered from different sources can be easily integrated.

Furthermore, the presented methodology is easy to implement, and, as it just employs FSI solutions in the construction of the database, it can be carried out using simulations performed with specific or general-purpose commercial software, making it flexible, and thus, suitable for the introduction in the clinical workflow. The method is more flexible than the use of Kalman filtering, which, unfortunately, is still not implemented in most of the FSI software packages, and is generally applied to in-house solvers, making it difficult to introduce in the clinical practice.

Despite these encouraging results, several questions remain unanswered. The first one is how to ensure that the measurements are enough to guarantee that the projection problem has a unique solution. The other is the selection of the snapshots, that is, the set of different simulations that form the database, to maximize the information extracted from the system with the minimum amount of work. Although this has been successfully solved for the study case shown in this work, future work is needed to generalize this parameter estimation technique for other different clinic problems, and to improve the characterization of the FSI model by using a more realistic model parameters and boundary conditions, especially for the solid regions.

## Footnotes

1 Note that the transformation $H$ can handle average measurements of any variable (i.e., velocity, wall displacement, stresses) or other variable available, either extracted as time signal or at a specific instant. The only restriction is that the set of measurements should contain enough information to ensure that the problem is well posed.

2 As these data have already been published as part of the CFD Challenge organized at STACOM 2013 [25], held in conjunction with MICCAI in September 2013, ethical board approval is not required.
